# Arsenic Accumulation and Physiological Response of Three Leafy Vegetable Varieties to As Stress

**DOI:** 10.3390/ijerph19052501

**Published:** 2022-02-22

**Authors:** Yuan Meng, Liang Zhang, Zhi-Long Yao, Yi-Bin Ren, Lin-Quan Wang, Xiao-Bin Ou

**Affiliations:** 1 College of Agriculture and Forestry, Longdong University, Qingyang 745000, China; txzzl891017@163.com (L.Z.); yzl8844@163.com (Z.-L.Y.); 18093486955@163.com (Y.-B.R.); 2 Gansu Key Laboratory of Protection and Utilization for Biological Resources and Ecological Restoration, Qingyang 745000, China; xbou@zju.edu.cn; 3 College of Resources and Environment, Northwest A&F University, Xiangyang 712100, China; linquanw@nwsuaf.edu.cn

**Keywords:** heavy metals, garland chrysanthemum, lettuce, antioxidant defense enzymes, GSH, PCs

## Abstract

Arsenic (As) in leafy vegetables may harm humans. Herein, we assessed As accumulation in leafy vegetables and the associated physiological resistance mechanisms using soil pot and hydroponic experiments. Garland chrysanthemum (*Chrysanthemum coronarium* L.), spinach (*Spinacia oleracea* L.), and lettuce (*Lactuca sativa* L.) were tested, and the soil As safety threshold values of the tested leafy vegetables were 91.7, 76.2, and 80.7 mg kg^−1^, respectively, i.e., higher than the soil environmental quality standard of China. According to growth indicators and oxidative stress markers (malondialdehyde, the ratio of reduced glutathione to oxidized glutathione, and soluble protein), the order of As tolerance was: GC > SP > LE. The high tolerance of GC was due to the low transport factor of As from the roots to the shoots; the high activity of superoxide dismutase, glutathione peroxidase, and catalase; and the high content of phytochelatin in the roots. Results of this work shed light on the use of As-contaminated soils and plant tolerance of As stress.

## 1. Introduction

Arsenic (As) is a non-metal, but as its toxicity and some of its properties are similar to those of heavy metals, it is generally included in the range of heavy metal pollutants [1]. The density of As is 5.727 g cm^−3^, and it is toxic and biologically non-essential. Many countries, including China, the United States, and Canada, have listed it as an environmental priority pollutant [2]. Direct exposure to As pollution will lead to liver poisoning and kidney damage, further causing hypertension, cardiovascular disease, and even cancer [3]. Environmental pollution such as heavy metal contamination has also been reported to increase the risk of mental disorders in humans [4]. There are even reports that the living environment and diet of captive giant pandas in China contain heavy metals, such as As and cadmium (Cd) [5]. In 2016, the State Council of China issued the “soil pollution prevention and control action plan”, which shows that China’s soil pollution control has become an increasingly urgent problem.

According to the bulletin of China’s national survey, inorganic pollutants are mainly responsible for soil pollution in China. Notably, the exceedance rate of soil As pollution points was 2.7%. It is difficult and impractical to eliminate heavy metal contamination in the soil [6,7]. Assessing how to reasonably and safely use contaminated soil has important theoretical and practical significance. Although the distribution area of heavy metal pollution is wide, the distribution of pollutants in the soil is shallow, and the degree of pollution is light. Thus, the cultivation of food or vegetable crops with a low-enrichment capacity is feasible [8,9]. Heavy metals in the environment enter the human body through the food chain. Vegetables are an indispensable food for human beings, being rich in vitamins, minerals, and crude fiber necessary for the digestive system. Leafy vegetables are believed to be more vulnerable to heavy metal pollution than other crops, such as *Solanaceae*, *Brassica oleracea*, root vegetables, shallots, and legumes [8,10,11]. They therefore have a higher potential safety risk, and thus their contamination is of great concern worldwide.

The mechanism of Cd enrichment and tolerance in the three leafy vegetables has been explored [12]. The mechanism of plant response to As is similar to that of Cd. Enzymes within the antioxidant system are responsible for scavenging reactive oxygen free radicals in cells caused by heavy metals, and the thiol pool is related to the compartmentalization of heavy metals in cells [13]. However, there are few reports on the response mechanism of garland chrysanthemum (GC), spinach (SP), and lettuce (LE) to As stress. This study aimed to compare the As bioconcentration factors of different leafy vegetables and reveal the associated mechanisms to provide a basis for the rational and effective utilization of heavy metal contaminated soil.

## 2. Materials and Methods

### 2.1. Soil Pot Experiment

**Figure 1:**
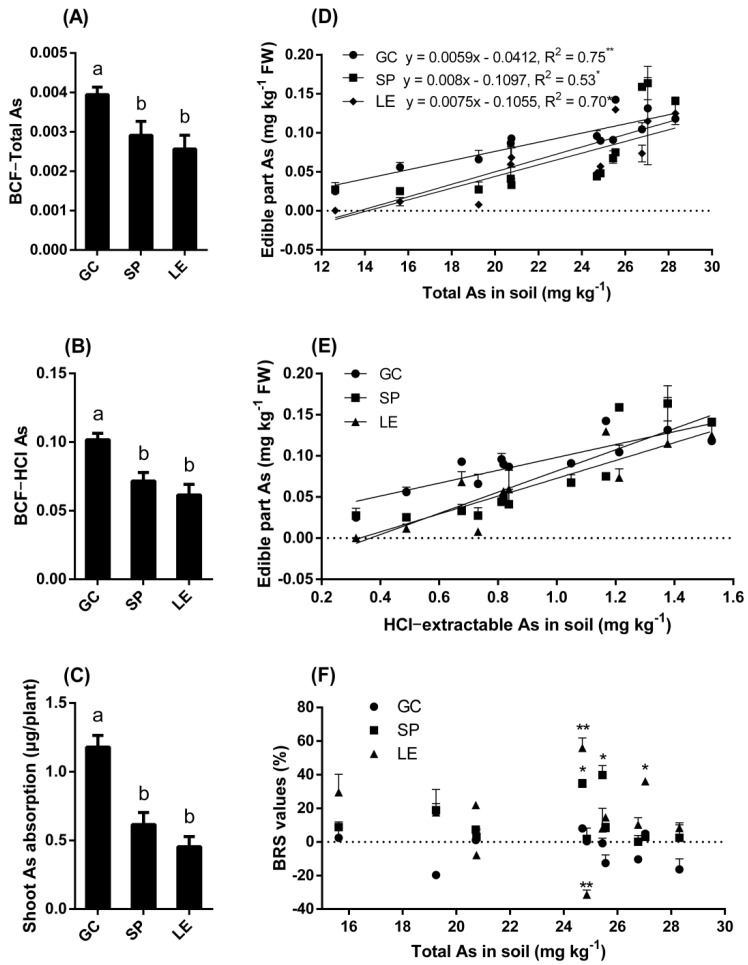
Bioconcentration factor (**A**,**B**), absorption (**C**), vegetable As concentration (**D**,**E**), and BRS values (**F**) in the soil test. Note: The different lowercase letters in the figure indicate significant differences between different vegetables (5% levels). The symbols * and ** indicate that the difference between values is significant at the respective 5% and 1% levels.

The pot experiment was completed under a shelter from 6 March to 25 April 2015. The shelter was ventilated, light-transparent, and rainproof, and its temperature was the same as the temperature outside. The vegetable seeds were purchased from the Shaanxi Hua-xing Green Seed Company (Yangling, Shaanxi 712100, China). On 6 March, spinach (*Spinacia oleracea* L.), lettuce (*Lactuca sativa* L.), and garland chrysanthemum (*Chrysanthemum coronarium* L.) were sown in black pots with a top diameter, bottom diameter, and height of 14, 10, and 12 cm, respectively. There were three small holes in the bottom of each pot, and the pots containing plant seeds were placed in a plastic tray to prevent soil erosion when watering. Water was added to the plastic tray to retain soil moisture during the experiment, and 1.6 g of basal fertilizer (15-15-15) was applied to each pot. Based on previous investigations of heavy metal pollution in soil, cultivated soil (0–20 cm) from vegetable fields with different degrees of pollution in the Baqiao District, Weiyang District, and Lintong District of Xi’an city (China) was collected. A total of 12 treatments (i.e., different As levels; as shown in Figure 1, the As concentration in the soil was 12.63–28.31 mg kg^−1^) were performed with three types of leafy vegetables (three repetitions) and 108 pots.

### 2.2. Hydroponic Test

The hydroponic test was carried out in a light incubator with a light intensity of 20,000 lx. The experiment took place with a photoperiod of 16 h of light (23 °C) and 8 h of darkness (15 °C). The seeds had been soaked in sterile water for 6–8 h prior to the experiment, then germinated in the seedling tray for about 10 days, and finally cultured with 1/10 modified Hogland solution. The hydroponic nutrient solution contained the following [14]: 0.1 mM NH_4_H_2_PO_4_, 0.505 mM KNO_3_, 0.15 mM Ca(NO_3_)_2_, 0.1 mM MgSO_4_, 1.64 µM FeSO_4_, 0.91 µM MnCl_2_, 0.03 µM CuSO_4_, 0.16 µM ZnSO_4_, 4.63 µM H_3_BO_3_, 0.06 µM H_2_MoO_4_, and 0.81 µM Na_2_EDTA. The hydroponic device was a cylindrical container with a diameter and height of 12 and 15 cm, respectively. The outside of the container was wrapped in black plastic to shade the roots. A sponge plate with two holes was placed above the hydroponic container to fix the leafy vegetables. The As concentrations of the hydroponic treatment (four repetitions) were 0 μM (0), 200 μM (As1), and 400 μM (As2). The compound used in the hydroponic test was sodium dihydrogen arsenate (Na_2_HAsO_4_). Vegetable seedlings were treated in the As solution on day 24 and harvested on day 31. Hydroponic As treatment was carried out for 7 d. The leafy vegetable samples were determined immediately after harvesting. The fresh samples were stored in liquid nitrogen for subsequent testing, whereas the dry samples were crushed for dry weight determination.

### 2.3. Assay Methods

Plant sampling method: For the soil pot experiment, when harvesting, scissors were used to destroy the pot, and the leafy vegetable roots and soil were taken out completely, soaked in a bucket and rinsed gently with water until all the plants and roots were clean. For the hydroponic experiment, the roots of vegetable plants were cut directly from the stem base and washed with deionized water 4 times. The method of removing metal ions attached to the root surface was to immerse the root in 20 mM Na_2_-EDTA for 15 min [15].

For the determination of chlorophyll content [16], 80% acetone was used to extract total chlorophyll (a, b) and carotenoids, and the contents were calculated by the formulas: chl a (μg/mL) = 12.7 OD_663_–2.69 OD_645_; chl b (μg/mL) = 22.9 OD_645_–4.68 OD_663_; car = 20.2 D_645_ + 8.02 D_663_.

For the determination of As content [15], the soil and vegetable samples were digested with aqua regia-perchloric acid and 10% nitric acid (HNO_3_). The soil-available arsenic was extracted with 0.1 mol·L^−1^ hydrochloric acid (HCl). The As content was determined using an inductively coupled plasma mass spectrometer (ICP-MS 7500, Agilent, Santa Clara, CA, USA).

The oxidative stress marker malondialdehyde (MDA) [17] was determined by the 0.5% thiobarbituric acid (TBA) method, which produces a reddish-brown color reaction with MDA in the tissue. Soluble protein was measured with Coomassie Brilliant Blue G-250.

To determine the plant superoxide dismutase (SOD), glutathione peroxidase (POD), and catalase (CAT) enzyme activities [16], the activities of SOD and CAT were quantified on a Shimadzu UV-2450 spectrophotometer (Japan) by measuring the ability to inhibit the photochemical reduction of nitroblue tetrazolium (NBT) and by monitoring the disappearance of H_2_O_2_ at 240 nm. The activity of POD was quantified by catalyzing the oxidation of guaiacol to brown products.

The determination of the thiol pool (cysteine (cys), γ-glutamylcysteine (γ-EC), reduced glutathione (GSH), and phytochelatins (PCs)) was as follows [18]: The determination of cys was measured by an acidic ninhydrin reagent at 560 nm. The γ-EC and PC determination method used mBBr to derivatize thiol, and γ-EC, PC_2_, and PC_3_ were used as standard materials for the determination of thiol by high-performance liquid chromatography (HPLC) coupled with parallel mass spectrometry for thiol-containing compounds in the plant samples. For the determination of GSH and GSSG, the absorbance was read at 412 nm by adding phosphate buffer (100 mM, pH 7.0) and 5′5-dithiobis-2-nitrobenzoic acid (DTNB) to the supernatant.

### 2.4. Standards and Calculation Formulas

The criteria for judging soil pollution were as follows: the ”Agricultural Land Soil Pollution Risk Screening Value“ (pH > 7.5, As: 25 mg kg^−5^; 6.5 < pH ≤ 7.5, As: 30 mg kg^−1^; 5.5 < pH ≤ 6.5, As: 40 mg kg^−1^; pH ≤ 5.5, As: 40 mg kg^−1^) according to China’s Soil Environmental Quality Standard (GB 15618-2018).

The calculation of the critical value of heavy metal safety was done as follows: First, the concentration of As in the edible vegetables (y, mg kg^−1^) and the concentration of As in the soil (x, mg kg^−1^) were used to fit the linear equation, and then the limit value of heavy metal in the leafy vegetables [the “Limits in Foods” standard (GB2762-2017) stipulates that the limit value of As is 0.5 mg kg^−1^ FW] was substituted into the equation. Finally, the obtained x value was the safety threshold of soil As.

$$\mathrm{BCF}1=\frac{a}{b}$$

where BCF_1_ is the bioconcentration factor of the soil total As; a is the concentration of As in the vegetable shoot (mg kg^−1^ FW); b is the total concentration of the soil As (mg kg^−1^).

$$\mathrm{BCF}2=\frac{a}{c}$$

where BCF_2_ is the bioconcentration factor of the soil HCl-extracted As; a is the concentration of As in the vegetable shoot (mg kg^−1^ FW); c is the soil HCl-extracted As concentration (mg kg^−1^).

$$\mathrm{TI}(\%)=\frac{\mathrm{DWt}}{\mathrm{DWck}}*100$$

where TI is aboveground/root/total tolerance index; DWt is the average dry weight of shoots/roots/total plant in the As treatment group; DWck is the shoot/root/total average dry weight in the control group.

$$\mathrm{BRS}(\%)=\frac{\mathrm{BIOt}-\mathrm{BIOck}}{\mathrm{BIOck}}*100$$

where BRS is biomass response to stress; BIOt is the biomass in the As treatment group; BIOck is the biomass of the control vegetable.

$$\mathrm{TF}=\frac{x}{y}$$

where TF is the transfer factor (i.e., the concentration ratio); x is the As concentration in the shoots; y is the As concentration in the roots.

$$\mathrm{Absorption}=\frac{d\times e}{1000}$$

where Absorption is the As absorption of each leafy vegetable (μg); d is the vegetable As content (mg kg^−1^); e is dry weight of the vegetable (mg).

$$\mathrm{SR}\;\mathrm{ratio}=\frac{g}{h}$$

where SR ratio (i.e., the accumulation ratio) is the accumulation ratio of shoot As to root As; g is As absorption (shoots); h is As absorption (roots).

### 2.5. Data Processing Methods

As shown in Figure 1D, the univariate linear regression was performed using SAS V8 software (North Carolina State University, Raleigh, NC, USA). ANOVA (analysis of variance) was performed, and Duncan’s test was used for multiple comparisons in the SAS program. As shown in Figure 1F, the ‘TTEST’ process of SAS software was run, which was used for the significance testing of the difference in unpaired data means. SPSS 20.0 (IBM Inc., Armonk, NY, USA) was used for the two-way ANOVA. GraphPad Prism6 (La Jolla, CA, USA) was used to construct the graphs.

## 3. Results

### 3.1. The As Stress Test of Leafy Vegetables in the Soil

The bioconcentration factor of GC was significantly higher than that of SP and LE (Figure 1A,B). The amount of As absorbed in the edible parts of GC was significantly higher than that of SP and LE (Figure 1C). Figure 1D,E show the relationship between the As content in the edible parts of the vegetables and the total As content in the soil or HCl-extracted As content, and there was a linear positive correlation between the two. The equation of the line is shown in Figure 1D. The heavy metal limit (0.5 mg kg^−1^ FW) was substituted into the equation. The calculated soil safety critical values of GC, SP, and LE were 91.7, 76.2, and 80.7 mg kg^−1^, respectively. Figure 1F shows the BRS values of the vegetables in the soil. When the As content in the soil was 24.86 mg kg^−1^, the BRS value of LE was −31.41%. When the As content in the soil was 24.69, 25.44, and 27.04 mg kg^−1^ (the respective soil organic matter content was 14.0, 13.1, and 11.0 g kg^−1^), the BRS values of SP and LE were positive, which may be due to the higher organic matter content in the soil.

**Figure 2:**
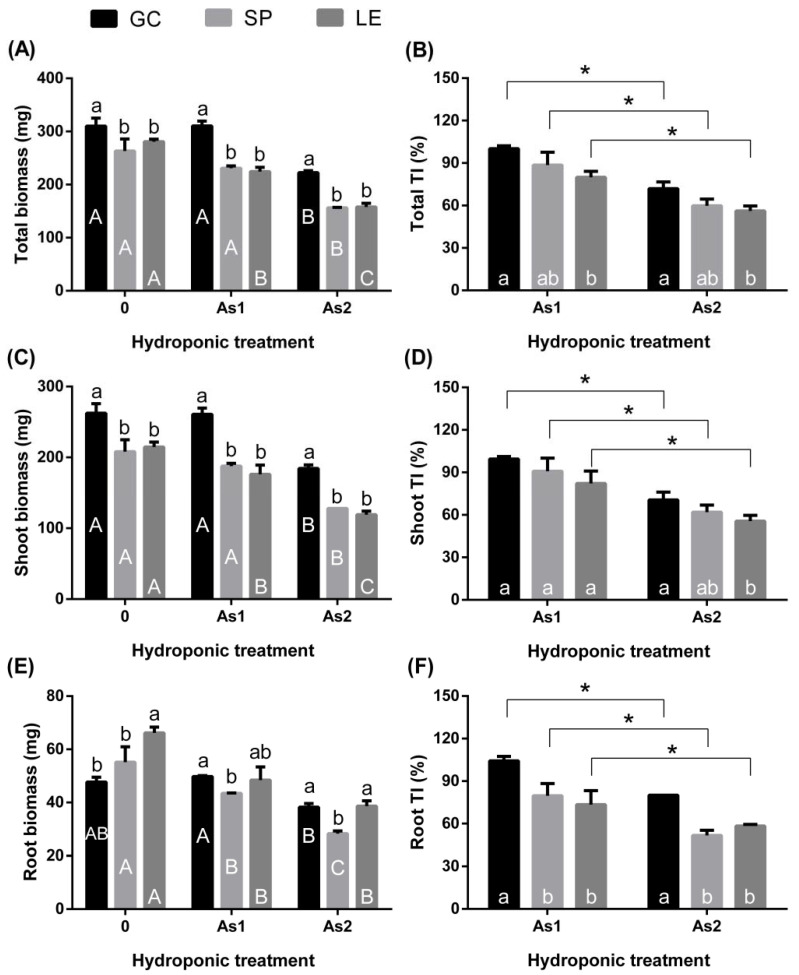
Biomass (**A**,**C**,**E**) of GC, SP, and LE and the TI of the plants to As in the hydroponic test (**B**,**D**,**F**). Note: Different lowercase letters in the figure indicate comparisons between different vegetables under the same treatment; different uppercase letters indicate the comparison of the same vegetable under different treatments (5% levels). The symbol * indicates that the difference in values between As1 and As2 was significant at the respective 5% levels. The error bars in the graph indicate the SEM (standard error of the mean).

The biomass of the vegetables showed a significant downward trend (Figure 2A,C,E) with the increase in As concentration in the hydroponic solution. However, under the As1 treatment, there was no significant change in the total biomass and the shoot and root biomass of GC compared to the CK treatment. The As TI of the whole plant, shoots, and roots of GC was significantly higher than that of SP and LE (Figure 2B,D,F). The TI of SP was slightly higher than that of LE, but not significantly so.

**Figure 3:**
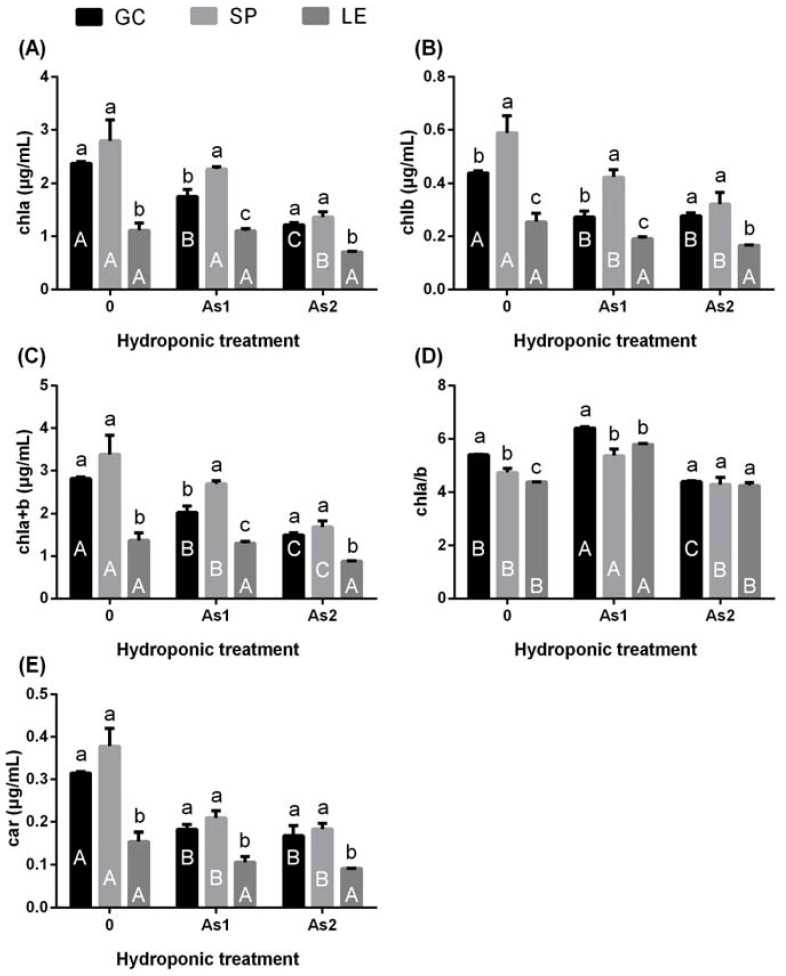
The content of chlorophyll (chla, chlb) (**A**,**B**) and carotenoids (car) (**E**) and the values of chla+b (**C**) and chla/b (**D**) of GC, SP, and LE under hydroponic As treatment. Note: Different lowercase letters in the figure indicate comparisons between different vegetables under the same treatment; different uppercase letters indicate the comparison of the same vegetable under different treatments (5% levels). The error bars in the graph indicate the SEM (standard error of the mean).

As shown in Figure 3, the chla, chlb, car, and chla+b of the vegetables decreased with the increase in As content in the hydroponic treatment (Figure 3A–C,E). The decrease in the chla, chlb, car chla/b, and chla+b content in LE was the smallest among all of the vegetables, and the results were not significant. It can be seen from Figure 3D that under the As1 treatment the chla/b value of the vegetables was significantly higher than that of CK and As2.

### 3.2. As Enrichment and Distribution in Plants

**Figure 4:**
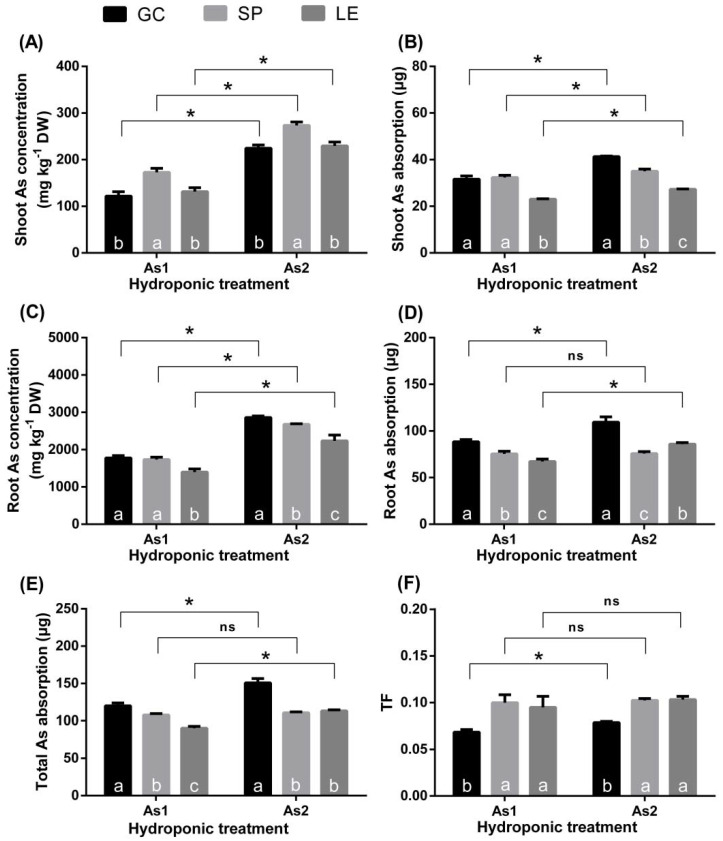
The As concentration (**A**,**C**), As absorption amount (**B**,**D**,**E**), and the root-to-ground TF of As (**F**) under hydroponic culture. Note: Different lowercase letters in the figure indicate comparisons between different vegetables under the same treatment (5% levels). The symbol * indicates that the difference between values is significant at the respective 5% levels. The error bars in the graph indicate the SEM (standard error of the mean).

As the concentration of As in the hydroponic solution increased, the As concentration in the shoots and roots of the vegetables increased (Figure 4A,C). The concentration of As in the shoots and roots of SP was high. By contrast, the concentration of As was low in the shoots of GC but high in the roots of GC. The As concentrations in the shoots and roots of LE were both low. The absorption of As by the shoots and roots of LE was very low, while that of GC was high (Figure 4B,D,E). Figure 4F shows the TF of As from the roots to the shoots of the vegetables. The TF of GC was the lowest, and there was no significant difference in TF between SP and LE.

### 3.3. Oxidative Stress Biomarkers and Antioxidative Defense Enzymes

**Figure 5:**
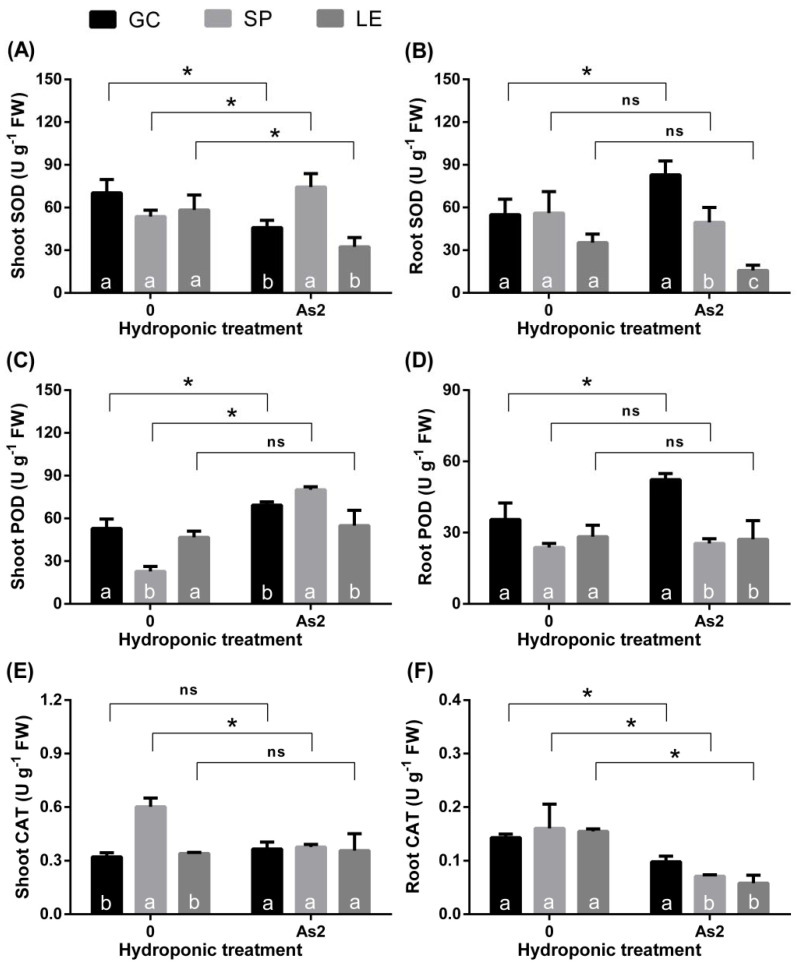
SOD (**A**,**B**), POD (**C**,**D**), and CAT (**E**,**F**) activities in the shoots and roots of GC, SP, and LE in the hydroponic As treatment. Note: Different lowercase letters in the figure indicate comparisons between different vegetables under the same treatment (5% levels). The symbol * indicates that the difference between values is significant at the respective 5% levels. The error bars in the graph indicate the SEM (standard error of the mean).

The SOD activity in the shoots of GC and LE decreased compared with CK, while there was an increasing trend in SP (Figure 5A). Under As treatment, the activity of SOD in the roots of GC increased, whereas SP and LE remained unchanged (Figure 5B). The As treatment increased the POD enzyme activity in the shoots of GC and SP, while that of LE remained unchanged (Figure 5C). The POD activity in the roots of GC increased, while that in SP and LE remained unchanged (Figure 5D). The activity of CAT in the shoots of GC and LE remained unchanged, while that in SP decreased significantly (Figure 5E). Compared with the CK treatment, the CAT activities of the roots of the three vegetables were significantly decreased (Figure 5F). Under As treatment, the activities of SOD (Figure 5A) and POD (Figure 5C) in the shoots of SP were significantly higher than those of GC and LE. The SOD activity of the roots of SP was higher than that of LE but lower than that of GC (Figure 5B). The activities of SOD, POD, and CAT in the roots of GC were the highest among the three vegetables (Figure 5B,D,F). Under As treatment, there was no significant difference in CAT enzyme activity in the aboveground parts of the three vegetables (Figure 5E,F).

**Figure 6:**
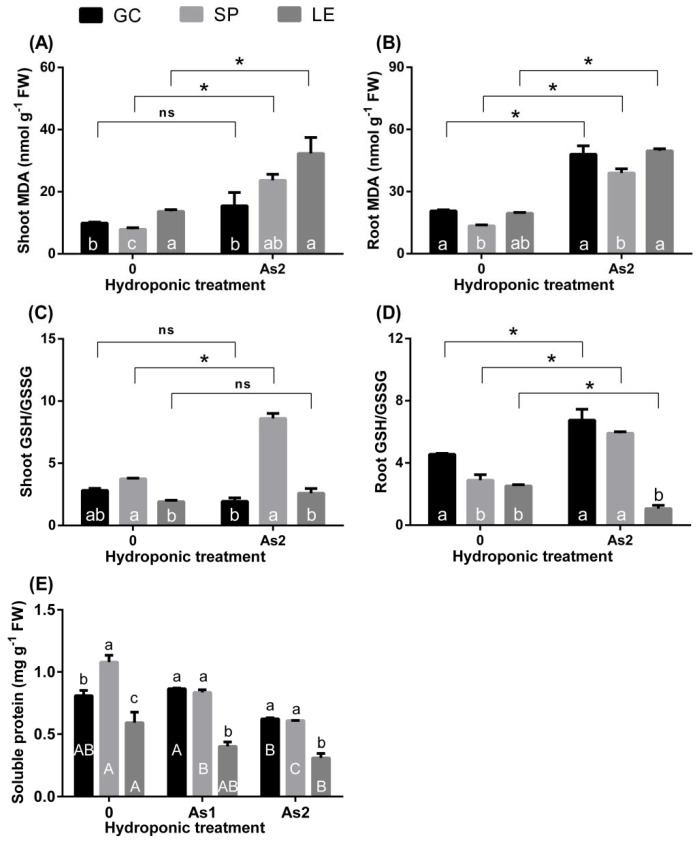
MDA (**A**,**B**), GSH/GSSG ratio (**C**,**D**), and soluble protein (**E**) content in the vegetables under hydroponic As treatment. Note: Different lowercase letters in the figure indicate comparisons between different vegetables under the same treatment; different uppercase letters indicate the comparison of the same vegetable under different treatments (5% levels). The symbol * indicates that the difference between values is significant at the respective 5% levels. The error bars in the graph indicate the SEM (standard error of the mean).

Figure 6 indicates that compared with CK the As treatment increased the MDA content in the shoots and roots of the vegetables (Figure 6A,B). The MDA content in the upper part of LE was significantly higher than that in GC, and the MDA content in the roots of SP was the lowest. Furthermore, under As treatment, the proportion of GSH/GSSG in the upper part of SP was significantly higher than that in the CK treatment (Figure 6C) and was significantly higher than that of GC and LE. The As treatment increased the ratio of GSH/GSSG in the roots of GC and SP and decreased the ratio of GSH/GSSG in the roots of LE (Figure 6D). The proportion of GSH/GSSG in the roots of LE was significantly lower than that in the other vegetables. The As treatment significantly reduced the content of soluble protein in the vegetables (Figure 6E), and the soluble protein content in LE was significantly lower than that in GC and SP.

### 3.4. Thiol Pool Concentration

**Figure 7:**
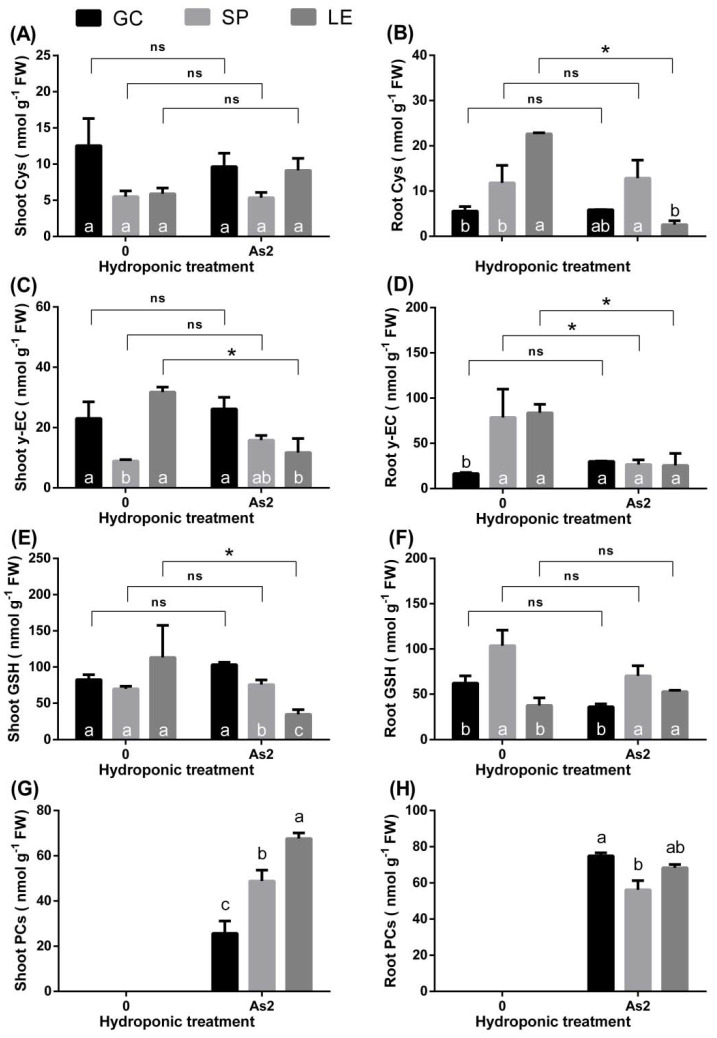
Content of thiol [cys (**A**,**B**), γ-EC (**C**,**D**), GSH (**E**,**F**), PCs (**G**,**H**)] in the shoots and roots of vegetables in the hydroponic experiments. Note: Different lowercase letters in the figure indicate comparisons between different vegetables under the same treatment (5% levels). The symbol * indicates that the difference between values is significant at the respective 5%. The error bars in the graph indicate the SEM (standard error of the mean).

Figure 7 shows the contents of the vegetable thiol pool (cys, γ-EC, GSH, and PCs). Under As treatment, the cys content in the shoots and roots of GC and SP remained unchanged compared to the control, while that in the roots of LE decreased (Figure 7A,B). The roots of LE had the lowest amount of cys. In the As treatment, the shoot γ-EC content of GC and SP remained stable, while that of LE declined markedly compared with the control (Figure 7C). The shoot γ-EC content of LE was lower than that of the other vegetables. There was a decreasing trend in the root γ-EC content of SP and LE under As exposure (Figure 7D). Compared with the treatment without heavy metals, the shoot GSH content of LE decreased under As stress (Figure 7E). By contrast, in the As treatment, there were no obvious changes (Figure 7F). The order of the PC content in aboveground parts was GC < SP < LE (Figure 7G). Differences were observed in the roots under As treatment, i.e., GC had a higher PC content (Figure 7H).

## 4. Discussion

### 4.1. Leafy Vegetables Suitable for Cultivation on As-Contaminated Soil

Figure 1 shows that the soil safety thresholds of As in GC, SP, and LE were 91.7, 76.2, and 80.7 mg kg^−1^, respectively, which are much higher than the soil environmental quality standard for agricultural land (25 mg kg^−1^). The safety critical value of heavy metals refers to when the concentration of heavy metals in the soil is higher than this value, indicating that the concentration of heavy metals in the edible part of leafy vegetables exceeds the limit value of the pollutants. Based on this, the three leafy vegetables planted on soil slightly polluted with As can be safely consumed. In the soil tests, after 50 days of cultivation of GC, SP, and LE on soil slightly polluted with As, the amount of As enriched in the plant was 1.18, 0.62, and 0.45, μg plant^−1^, respectively (Figure 1). The World Health Organization (WHO) stipulates that the allowable intake of inorganic As for adults is about 128 μg person^−1^ D^−1^. For children aged 0–6 years, the short-term and long-term As exposure should be less than 15 and 5 μg person^−1^ D^−1^ [19]. According to this regulation, as long as the daily number of GC, SP, and LE that is consumed does not exceed 108, 206, and 284, the health of adults will not be harmed by the consumption of these leafy vegetables. For children, the leafy vegetable intake number limits of short-term As exposure of GC, SP, and LE are 13, 24, and 33, respectively, while the limits for long-term As exposure are 4, 8, and 11, respectively. This assumes that cooking does not affect the As content in the vegetables; that is, the As content in vegetables is equal to the amount of As ingested by the human body through the vegetables.

### 4.2. Tolerance Metabolism

#### 4.2.1. As Tolerance of the Leafy Vegetables

GC can endure As stress better than SP and LE. This was confirmed by our hydroponic experiment results. Growth [20] and oxidative stress [21] are two tolerance criteria widely accepted by researchers. Furthermore, oxidative stress markers (MDA, GSH/GSSG, and soluble protein) can measure the degree of oxidative stress [22,23]. MDA reflects the degree of cell membrane lipid peroxidation, while GSH/GSSG is an indicator of cellular redox potential [24]. Accordingly, the higher the proportion of GSH/GSSG, the higher the ability of plants to scavenge reactive oxygen species in the cells [25]. LE had the highest MDA content and the lowest GSH: GSSG in both the shoots and roots. The content of its soluble protein was much lower than those of the other leafy vegetables. These findings indicate that the degree of oxidative stress caused by As in LE was the highest among the three vegetables, and thus LE is an As-sensitive vegetable. As for GC, its root and shoot TI was the highest among the three vegetables (Figure 2), indicating that it is an As-tolerant variety.

#### 4.2.2. Absorption and Transport of As in Plants

In the hydroponic experiment, the absorption of As in the whole, aboveground, and root parts of GC was the highest among the three vegetables (Figure 2). In addition, the soil pot experiment also confirmed that the As bioconcentration factor and the amount of As in the edible parts of GC were higher than those of SP and LE (Figure 1C,E). More As entering GC cells means more damage [21]. However, GC had the highest As TI. This means that the As uptake and accumulation ability of plants does not completely determine the tolerance of plants [20,24]. This situation is also clearly reflected in LE, wherein the concentration and absorption of As in the shoots and roots of LE were at the lowest level, but its TI was the lowest among the three vegetables.

The canopy is the direct foundation of plant growth because the leaves are the main organs of photosynthesis and provide the energy necessary for the growth of the entire plant. Additionally, roots are an important line of defense for heavy metals in the soil. The tolerance of plants to heavy metals is highly related to the transport of As to the shoots (TF). The As TF from the roots to shoots of GC was significantly lower than that of SP and LE (Figure 4). Similar conclusions were found in a study on *Oenothera odorata* [21].

#### 4.2.3. Enzyme Activity of the Antioxidant System

Heavy metal ions usually cause harm by interfering with or replacing the function of essential nutrient elements in plants [26] or causing the accumulation of reactive oxygen radicals in cells [25]. Enzymes of the antioxidant system can effectively alleviate the accumulation of reactive oxygen species; therefore, a higher enzyme activity of the antioxidant system leads to a stronger tolerance of As in leafy vegetables. SOD is the first line of defense for converting O_2_^−^ to H_2_O_2_, while POD and CAT are mainly responsible for the clearance of H_2_O_2_ in cells [25,26]. Under Cd stress, the tolerance of different leafy vegetables is related to the change of antioxidant enzyme activity, while the As tolerance is more related to the value of antioxidant enzyme activity. This shows that the antioxidant system of leafy vegetables has different responses to Cd and As. The specific reasons and underlying mechanisms need to be further studied. Under Cd stress, the activity of superoxide dismutase, peroxidase, and catalase in the roots of LE decreased significantly. Nevertheless, these parameters in the roots of SP and LE remained steady or were even enhanced [12]. In this experiment, under As stress, the roots of GC had the highest SOD, POD, and CAT activities (Figure 5), which improved the As tolerance of this vegetable. By contrast, the LE plants had the lowest enzyme activity in both the roots and shoots. The activities of SOD and POD in the shoots of SP were higher, whereas those in the roots were lower. This indicates that its As tolerance is higher than that of LE.

#### 4.2.4. Thiol Pool

Studies have shown that plants can alleviate heavy metal stress by immobilizing heavy metals in cell vacuoles through partitioning [26]. Plants usually produce a variety of chelating agents (PCs) to mask and reduce toxicity [13,27]. It is believed that As in plant cells is sequestered by GSH and PCs and fixed in the vacuoles for detoxification purposes [1,13,28]. In addition, the content of PCs (heavy metal chelators) can explain the difference in the As tolerance of vegetables to some extent. It appears that the high As tolerance of GC was due to its high content of PCs and their ability to chelate and fix As in the roots; thus, the transport of As from the roots to the shoots was reduced. Studies have found that heavy metal sensitive plants sequester heavy metals in the leaves rather than in the roots [29]. Our study found that the content of PCs was high in the shoots of LE but very low in the roots. However, the superiority of shoot PCs in LE did not alleviate the growth inhibition caused by As. A similar conclusion was drawn in the study of white lupin [30]. In this study, the PC content of the roots of SP were lower than that of L, which does not explain its higher tolerance. However, there is research on *O. odorata* suggesting that PC content is significantly positively correlated with As concentration in the roots [21]. These contradictory results indicate the diversity of plant resistance to heavy metal stress, which appears to partly depend on the plant variety.

In this study, the concentrations of GSH and PCs in the leafy vegetables showed the opposite trend, i.e., the higher the content of GSH, the lower the content of PC (Figure 7E–H). The decrease in the GSH concentration may be due to the synthesis of more PCs. Heavy metal induces the expression of intracellular phytochelatin synthase and other related genes [31] and forms a thiol pool according to the following sequence cys→γ-EC→GSH→PCs [1,13]. Research has found that the high content of the thiol pool induced by high SP treatment in the roots increased As accumulation [15]. Under As treatment, the content of cys in the roots of LE exhibited a downward trend, and there were no significant changes in the content of cys in the roots and shoots of the other leafy vegetables (Figure 7A,B). This finding shows that the content of cys in the leafy vegetables was sufficient, and As does not cause a big change. Under As treatment, the y-EC content in the shoots and roots of GC did not fluctuate significantly, while the y-EC content in the roots of LE and SP decreased (Figure 7C,D). This may be because GC has more abundant y-EC than SP and LE for the synthesis of GSH.

## 5. Conclusions

Screening for leafy vegetable species and varieties suitable for soils slightly polluted with heavy metals is beneficial for soil utilization. The present results showed that GC, SP, and LE can be planted in soil slightly polluted by As and can be consumed safely. The soil safety thresholds of As in GC, SP, and LE were 91.7, 76.2, and 80.7 mg kg^−1^, which are much higher than the soil environmental quality standard in agricultural land. GC is As tolerant, and LE is As sensitive. In addition, both the antioxidant enzyme system and thiol pool were found to play an important role in the As stress of leafy vegetables.

## Data Availability

Not applicable.
